# Irisin Enhances Osteoblast Differentiation *In Vitro*

**DOI:** 10.1155/2014/902186

**Published:** 2014-03-04

**Authors:** Graziana Colaianni, Concetta Cuscito, Teresa Mongelli, Angela Oranger, Giorgio Mori, Giacomina Brunetti, Silvia Colucci, Saverio Cinti, Maria Grano

**Affiliations:** ^1^Department of Basic Medical Science, Neuroscience and Sense Organs, University of Bari, 70124 Bari, Italy; ^2^Department of Clinical and Experimental Medicine, University of Foggia, 71100 Foggia, Italy; ^3^Department of Experimental and Clinical Medicine, Center of Obesity, United Hospitals—University of Ancona, 60020 Ancona, Italy

## Abstract

It has been recently demonstrated that exercise activity increases the expression of the myokine Irisin in skeletal muscle, which is able to drive the transition of white to brown adipocytes, likely following a phenomenon of transdifferentiation. This new evidence supports the idea that muscle can be considered an endocrine organ, given its ability to target adipose tissue by promoting energy expenditure. In accordance with these new findings, we hypothesized that Irisin is directly involved in bone metabolism, demonstrating its ability to increase the differentiation of bone marrow stromal cells into mature osteoblasts. Firstly, we confirmed that myoblasts from mice subjected to 3 weeks of free wheel running increased Irisin expression compared to nonexercised state. The conditioned media (CM) collected from myoblasts of exercised mice induced osteoblast differentiation *in vitro* to a greater extent than those of mice housed in resting conditions. Furthermore, the differentiated osteoblasts increased alkaline phosphatase and collagen I expression by an Irisin-dependent mechanism. Our results show, for the first time, that Irisin directly targets osteoblasts, enhancing their differentiation. This finding advances notable perspectives in future studies which could satisfy the ongoing research of exercise-mimetic therapies with anabolic action on the skeleton.

## 1. Introduction

The benefits of exercise have been widely recognized, indeed the physical activity is reported as the better nonpharmacological treatment for cardiovascular, metabolic, and bone diseases [1, 2]. However, for long time, the molecular mechanisms by which exercise exerts its healthful effects remained mostly unknown. A successful deal for researchers and clinicians should be to reveal these mechanisms, encouraging practicing physical activity and promoting the development of exercise-mimetic drugs.

Recently, several lines of evidence are suggesting that skeletal muscle is crucial in the regulation of energy homeostasis. Therefore, the skeletal muscle is now considered an endocrine organ that secretes a number of myokines including the newly identified Irisin [3]. In this work, Boström and colleagues have reported that physical exercise activity induces an increase of the transcriptional regulator Peroxisome Proliferator-Activated Receptor-*γ* Coactivator 1*α* (PGC-1*α*) in the skeletal muscle, which in turn drives the production of the membrane protein Fibronectin type III domain-containing protein 5 (FNDC5). This is subsequently cleaved as the myokine Irisin, which acts on white adipose tissue (WAT), stimulating uncoupling protein 1 (UCP1) expression, one of the master genes of brown adipose tissue (BAT), and activating the browning response [3]. The authors showed that, after 3 weeks of free wheel running, plasma Irisin levels in mice were increased by 65% and, in healthy humans, plasma Irisin levels were found to double after 10 weeks of endurance exercise [3]. These results opened new frontiers for searching the involvement of Irisin in a broader network, suggesting the intriguing feasibility that this myokine might represent an endocrine molecule that could target other organs besides the adipose tissue.

Notably, muscle is important for bone healing and activity. Indeed, the hypothesis that muscle supports bone mass is confirmed by studies of microgravity and bed rest [4], as well as results showing the associated development of sarcopenia and osteoporosis at the same time [5].

Physical exercise is fundamental for the development of an efficient weight-bearing skeleton. For instance, the exercise strengthens bones, given the evidence that tennis players develop high bone mass in the playing arm compared to the nonplaying arm [6]. On the other hand, the absence of physical exercise or, even worse, the complete disuse of muscles, ensuing by severe pathological conditions, leads to a likewise severe bone loss. For instance, child growing with congenital neuromuscular diseases develop fragile long bones with reduced periosteal circumference [7]. Furthermore, long bones in a paralyzed limb do not achieve their normal ossification and show delay of mineralization in newly laid-down bone matrix [8]. Consequently, this paralytic phenotype, associated with decreased bone mass, is prone to severe fractures as frequently occurs in progressive disease such as spina bifida [8].

Moreover, the propensity of sarcopenic patients to falls can often degenerate into an osteoporotic hip fracture. This event may reduce the life expectancy of up to two years and this corresponds to increased mortality of 20–25% in the first year after the fracture [2]. For this reason, the onset of simultaneous sarcopenia and osteoporosis, also described as “the hazardous duet,” has been defined as one of the most devastating threats during old age [2].

Although this tight relationship between skeletal muscle and bone is well recognized, it has hitherto been mainly explained concerning mechanical loading, as overall effect [9]. Therefore, the description of bone response to mechanical load is currently defined as the ability of bone cells to perceive paracrine signals produced by mechanical stimulus [10]. This mechanotransduction effect is highly anabolic in bone. Hence, it could be extremely useful to deepen the understanding of the molecular mechanism involved, revealing the identity of all these signalling-paracrine molecules able to affect bone metabolism. For this reason, we propose a model where exercise induces muscle to release myokines, which regulate mechanotransduction in bone, basing on the physical proximity of these two tissues. Noteworthy, we postulated that the protective effect of muscles on bone could be dependent on the paracrine action of the myokine Irisin. To validate the potential role of Irisin on bone metabolism, we investigated whether Irisin targets bone cells directly, demonstrating its ability to increase the differentiation of bone marrow stromal cells into mature osteoblasts.

The relevance of these findings opens new frontiers in searching the Irisin mechanism of action on bone metabolism. The *in vivo *data, confidently obtained in future, could further correlate the well-known beneficial effects of physical exercise with bone recovery and improvements.

## 2. Materials and Methods

### 2.1. Materials

Antibody anti-FNDC5 (Irisin cleaved form) was from Abcam; Antibody anti-Collagen I and *β*-Actin were from Santa Cruz, Antibody anti-*β* -Tubulin was from OriGene Technologies. Ascorbic acid, b-Glicerophosphate and Alkaline Phosphatase (ALP) staining kit were from Sigma Aldrich. Primers for qPCR are ALP/S-aaacccagacacaagcattcc; ALP/AS-tccaccagcaagaagaagcc; Coll I/S-ggctcctgctcctcttag; Coll I/AS-acagtccagttcttcattgc.

### 2.2. Exercise Protocol

2-month-old C57BL/6 male mice were subjected to 3 weeks of rest activity or free wheel running activity, as described previously [3]. The rest activity was performed isolating each mouse in one cage, in order to avoid their tendency to fight with cage mates, preventing any exercise-mimetic activity. The wheel mice were individually housed, in order to avoid that the dominant mouse in the cage inhibited other mice in the free use of wheel. Animals were euthanized by cervical dislocation and their tissues were surgically excised.

### 2.3. Primary Cell Cultures

Primary myoblasts were obtained from digestion of vastus lateralis specimens with a solution of trypsin, collagenase, and CaCl_2_. The isolated cells were preplated on an uncoated petri dish for 1 hour to remove fibroblasts and then transferred on tissue culture plate and cultured with *α*-MEM/10% FCS. Cells were then cultured for 14 days until multinucleated, spontaneously contracting myotubes were formed. After 3 days, the conditioned media (CM) were collected. Firstly, CM were centrifuged at 1300 rpm to eliminate floating cells. Then, CM were purified by centrifugation at 13 K rmp to eliminate debris.

Bone marrow stromal cells, obtained by flushing bone marrow of 2-month-old C57BL/6 mice, were cultured to induce osteoblast differentiation with *α*-MEM/5% FCS in the presence of 50 *μ*g/mL ascorbic acid and 10^−2^ M *β*-glycerophosphate or with 1/2 CM from primary myoblast +1/2*α*-MEM/10% FCS in the presence of 50 *μ*g/mL ascorbic acid and 10^−2^ M *β*-glycerophosphate. Thereafter cells were subjected to alkaline phosphatase staining and mRNA and protein analysis.

### 2.4. RT-PCR

qPCR was carried out after RNA extraction using spin columns (RNasy, Qiagen) according to the manufacturer's instruction. By using SuperScript First-Strand Synthesis System kit (Invitrogen), the resulting cDNA (20 ng) was subjected to quantitative PCR and, thereafter, to ITAQ SYBR Green Supermix with ROX kit (Bio-Rad) on an iCycler iQ5 Cromo4 (BioRad). Each transcript was assayed 3 times, and cDNA was normalized to murine Gapdh, 18S or *β*-Actin and quantitative measures were obtained using the ΔΔC_T_ method. Analyses were performed using unpaired Student's *t*-tests (Excel) for significant differences at *P* < 0.05.

### 2.5. Western Blot

Protein amounts from all samples were assessed using the BCA-kit (Biorad) followed by protein concentration normalization before all western blot experiments. 30 *μ*g of cell proteins was subjected to SDS-PAGE. Subsequently proteins were transferred to nitrocellulose membranes (Hybond, Amersham). The blots were probed using primary antibodies, described in Materials section, and IRDye-labeled secondary antibodies (680/800 CW) (LI-COR Biosciences). For immunodetection, the Odyssey infrared imaging system was utilized (LI-COR Corp., Lincoln, NE). All data were normalized to background and loading controls.

## 3. Results

### 3.1. Myoblast from Exercised Wheel Mice Express Higher FNDC5/Irisin Than Rest Mice

**Figure 1:**
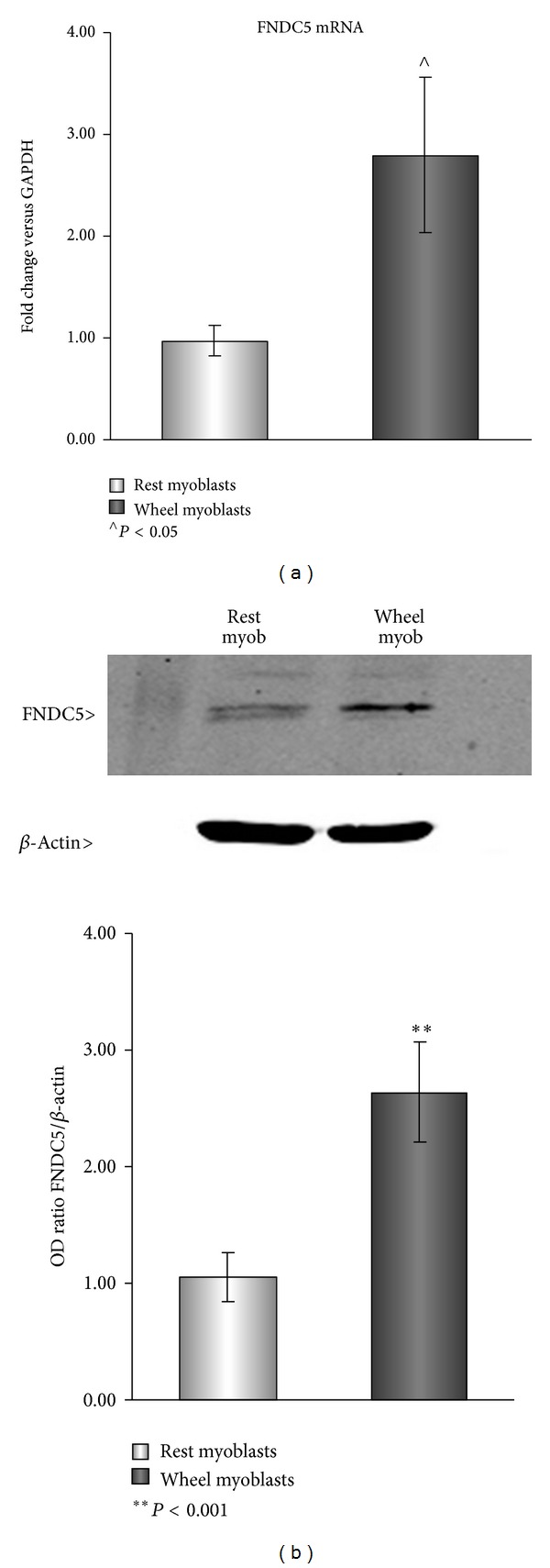
qPCR analysis of FNDC5 in mRNA extracts (a) and western blot analysis of Irisin/FNDC5 in total cell lysates (b) from primary culture of myoblasts obtained from mice subjected to 3 weeks of rest activity or free wheel running activity. *N* = 8 for each group, repeated in 3 separate experiments. Data is presented as mean ± SEM.  **P < 0.001 and ^∧^ *P* < 0.05 compared to rest group. Student's *t*-test was used for single comparison.

FNDC5 is highly expressed in skeletal muscle [3, 15]. Therefore, we confirmed the effects of exercise on FNDC5 expression in our exercise regimen, based on 3 weeks of voluntary free wheel running. By qPCR analysis, we detected a 2-fold increase in FNDC5 mRNA of wheel myoblasts (Figure 1(a)). This result was confirmed by the analysis of FNDC5/Irisin protein expression (Figure 1(b)). Indeed, we were able to detect a stronger band, corresponding to FNDC5/Irisin, in cell lysates of myoblasts from wheel mice compared with those from rest mice (Figure 1(b)). These data are, according to previous observations, showing an increase of about 65% in muscle of mice subjected to three weeks of voluntary exercise [3]. It should be further noted that FNDC5/Irisin is slightly detectable also in rest myoblasts, suggesting a constitutive expression of this myokine even in nonexercised state that might be related to a basal metabolism.

### 3.2. Conditioned Medium from Wheel Myoblasts Enhances Osteoblast Differentiation

**Figure 2:**
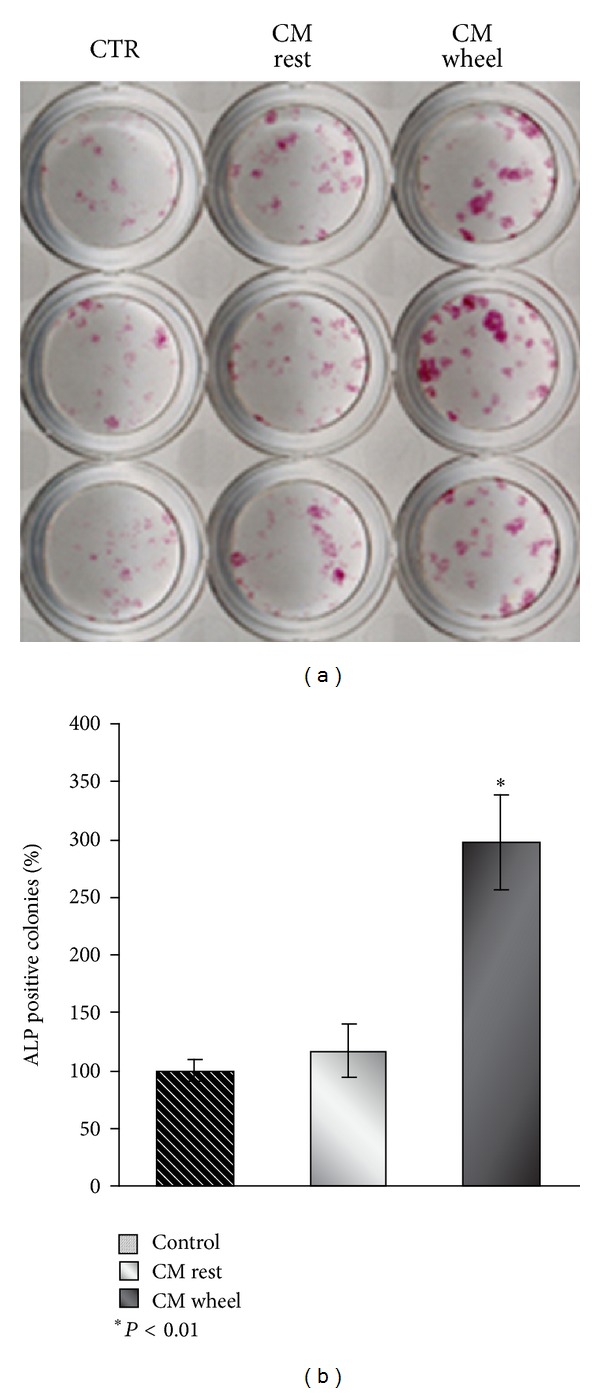
Histochemical staining for ALP in osteoblasts primary culture obtained from mouse bone marrow stromal cells treated with *α*-MEM/5% FCS in the presence of 50 *μ*g/mL ascorbic acid and 10^−2^ M *β*-glycerophosphate (CTR) or with 1/2 CM from primary myoblast (rest or wheel) +1/2*α*-MEM/10% FCS in the presence of 50 *μ*g/mL ascorbic acid and 10^−2^ M *β*-glycerophosphate. The graph shows quantification of ALP positive colonies as percentage (*P < 0.01) compared to control and is representative for 3 independent experiments. Data is presented as mean ± SEM. Student's *t*-test was used for single comparisons.

In the last years, accumulating evidences have shown that skeletal muscle release hormone-like substances. These secreted proteins are largely myokines and play important regulatory role in intercellular communication [16]. Our model of primary murine skeletal muscle cells allowed us to recapitulate *in vitro* what occurs *in vivo* when muscle is subjected to exercise and releases these circulating myokines. The conditioned medium (CM) collected from these myoblasts, which likely contained several released myokines, was used to evaluate its ability in regulating maturation of undifferentiated bone marrow stromal cells toward osteoblast differentiation. Our result shows that the CM obtained from wheel myoblasts increased by 2.5-fold the number of alkaline phosphatase (ALP) positive colonies compared to control medium (Figure 2). This assay, based on a histochemical staining for ALP, is the first evidence of osteoblast differentiation, since the ALP enzyme is established as the osteoblastogenesis relevant marker. The result suggests, for the first time, that muscles could exert a direct anabolic effect on osteoblasts through the paracrine action of released myokines, rather than the solely mechanotransduction action on osteocytes, the mechanosensor cells of bone.

### 3.3. Irisin Secreted from Wheel Myoblasts Increases Alkaline Phosphatase and Collagen I Expression

**Figure 3:**
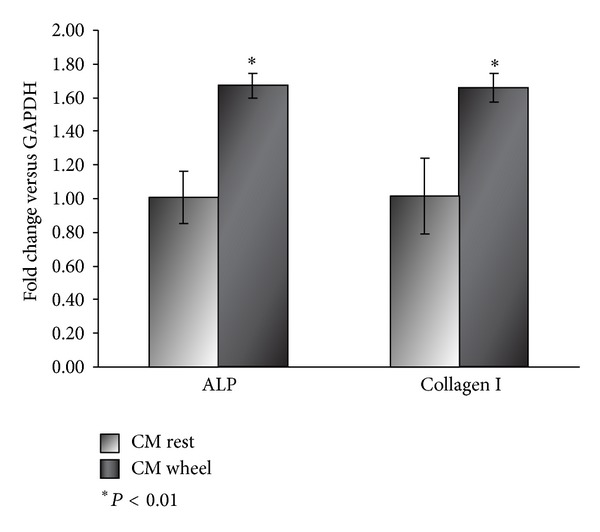
qPCRanalysis of ALP and Collagen I in mRNA extracts from osteoblasts treated with conditioned medium (CM) of myoblasts from rest and wheel mice. *N* = 8 for each group, repeated in 3 separate experiments. Data is presented as mean ± SEM. *P < 0.01 compared to rest CM. Student's *t*-test was used for single comparisons.

**Figure 4:**
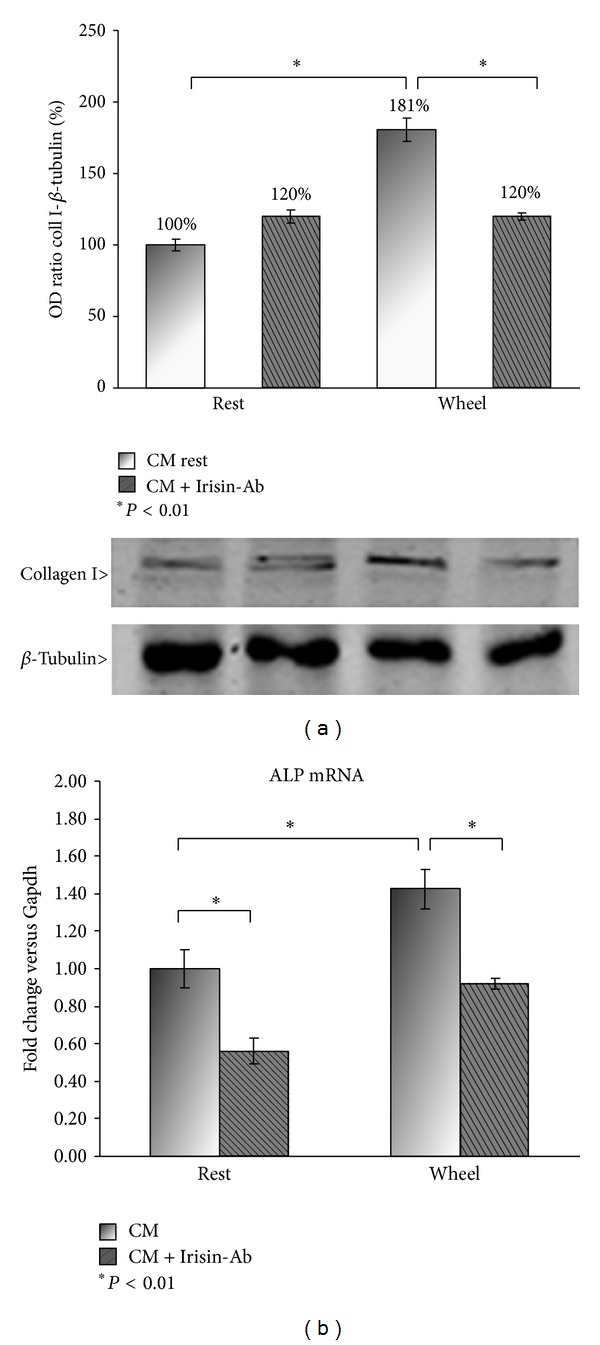
Western blot analysis of Collagen I in total cells lysates (a) and qPCR analysis of ALP in mRNA extracts (b) from osteoblasts treated with conditioned medium (CM) of myoblasts from rest and wheel mice ± Irisin/FNDC5 neutralizing antibody. The graph (a) shows quantification of OD ratio Collagen I/*β* -Tubulin as percentage (*P < 0,01) compared to rest CM and is representative for 3 independent experiments. Data is presented as mean ± SEM. Student's *t*-test was used for single comparisons.

Given the ability of CM from wheel myoblasts to enhance osteoblast differentiation, we investigated which bone proteins were upregulated. By qPCR analysis, we demonstrated that osteoblasts treated for 3 days with CM from wheel myoblasts have an increased expression of ALP and Collagen I mRNA (Figure 3). These data, further confirming the previously shown increased number of ALP positive colonies (Figure 2), is supported by an enhanced expression of ALP mRNA, the marker gene of osteoblasts. Moreover, the upregulation of Collagen I, the most abundant bone protein, greatly corroborates the beneficial effect on osteoblasts exerted by molecules released from the exercised muscle. Subsequently, our effort has been to obtain evidence about the involved myokine, present in CM, responsible for such a great effect on osteoblast differentiation. Given the increased expression of FNDC5/Irisin seen in myoblasts from wheel mice (Figures 1(a) and 1(b)), we choose Irisin as candidate myokine. For this challenge, we cultured osteoblasts with CM from myoblasts in presence of a neutralizing antibody direct against Irisin. We showed that the increase in Collagen I and ALP was completely reversed by neutralizing Irisin in wheel CM used to treat osteoblasts (Figures 4(a) and 4(b)). This finding demonstrated that the enhanced osteoblastogenesis, induced by exercised muscle, is Irisin-dependent.

It should be noted that the molecular weight of the secreted form of FNDC5/Irisin has remained for long time controversial. Now it is well ascertained that the sequence of mouse FNDC5 is cleaved from aa 29 to aa 151 to give its released form, as Irisin. Being aware of this, we used an anti-FNDC5/Irisin antibody (amino acids 50–150 from Abcam) directed against the predicted Irisin cleaved form.

## 4. Discussion

Boström and colleagues have recently reported that physical exercise activity induces the release, from skeletal muscle to bloodstream, of the myokine Irisin which was so called (from Iris, the messenger goddess) to highlight its role as positive messenger which targets an endocrine signal from skeletal muscle to adipose tissue (WAT). Irisin induces browning of WAT (i.e., conversion from WAT to BAT), that is a well-known new avenue for its great therapeutic potential in diabetes and obesity [3].

In the present study, we show that mature exercised primary myoblasts and myotubes secrete factor(s), which increase osteoblast differentiation *in vitro*. We also establish that this enhanced osteoblastogenesis, induced by exercised muscle, is mediated through Irisin. These results presented here add new insights in the complex relationship between muscle and bone tissue, indeed the mechanical influence of skeletal muscle on bone has long been documented but the molecular mechanisms involved remain still poorly understood [2]. Skeletal muscle and bone are tightly connected: they have a common origin, share the same integrated system that provides shape and physical function, and display significant changes across the lifespan. In elderly, the severe decline of skeletal muscle function, known as Sarcopenia, is associated with impaired function of bone (osteopenia). These two simultaneous losses of function lead to increased risk of falls and bone fractures. Therefore, developing a better understanding of the complex relationship between these important components of the musculoskeletal system may reveal new strategies for early identification, prevention, and treatment of sarcopenia and osteopenia, as well as their consequences [2, 8, 17, 18].

Currently, the most effective measure to counteract both diseases is exercise [1], but not all patients can perform a physical exercise program; thus, our evidence that exercise-induced Irisin could account for this effect greatly improves the chances of achieving this goal.

We show here that Irisin directly targets osteoblasts, enhancing their differentiation *in vitro*, proving that myokines, produced by exercised muscle, might be among the molecules regulating mechanotransduction in bone. In our system, mice subjected to three weeks of voluntary exercise showed an increased expression of Irisin/FNDC5 in skeletal muscles.

This result confirms previously published data demonstrating that endurance exercise training for 10 weeks increased plasma Irisin levels in healthy adult [3]. Conversely, Timmons et al. were not able to confirm FNDC5 gene activation by aerobic exercise in younger subjects [19]. These discrepancies have been explained by another study, which demonstrated that Irisin levels increase only when more energy is needed, such as in circumstances where ATP concentration in muscle is strongly decreased [20].

Moreover, we achieved evidence that conditioned medium from primary culture of myoblasts and myotubes, obtained from exercised muscles, were able to enhance the number of alkaline phosphatase (ALP) positive colonies in culture of undifferentiated bone marrow stromal cells.

Noteworthy, this is the first study showing the osteogenic potential of Irisin released from exercised skeletal muscle. From a physiological point of view, this result adds another explanation of the tightly connection between muscle and bone, which share a common fate even in positive scenarios, like in this concomitant gain of mass.

The effects of physical exercise are systemic and, obviously, cannot be solely related to the energy expenditure in muscle [21]. The study of Böstrom et al. [3] reported a new mechanism that explains how the total body energy expenditure is increased by muscle activity, elucidating the molecular circuit triggered by exercised muscle. Analysis of subcutaneous fat tissue depots, in mice overexpressing the muscle-specific PGC-1*α*, revealed that white adipocytes displayed signatures of brown fat cells [3]. Delineating muscle genes activated by PGC-1*α*, authors identified the myokine Irisin, able to drive the white-to-brown adipocyte transdifferentiation [3, 22].

By considering the tight relationship between skeletal metabolism and energy homeostasis, clinical and experimental results are giving great importance to the role of BAT on bone metabolism. Due to the evidence that an inducible form of BAT exists during adulthood and given the importance of its ability to dissipate the stored energy with thermogenesis [23–26], BAT induction is becoming a significant promise for the treatment of obesity and metabolic syndrome [13]. A cross-sectional study, carried on 15 young women, has shown a positive correlation between the amount of BAT and bone mineral density (BMD) [27]. Data derived from experimental mice model showed that, FoxC2(AD)(+/Tg) mice, overexpressing FoxC2 as well-established model for induction of BAT have high bone mass due to increased bone formation associated with high bone turnover [28]. On the contrary, mice named Misty (m/m), carrying a very low amount of BAT, albeit partially functional, have accelerated age-related trabecular bone loss and impaired brown fat function, such as reduced temperature and lower expression of PGC-1*α* [29].

**Figure 5:**
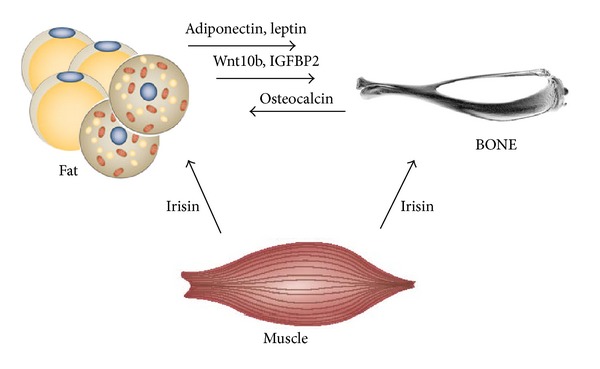
The muscle-fat-bone axis. It is been ascertained that a local network exists between the adipose and the bone tissue, which creates what has been defined as the fat-bone axis. In this paracrine circuit, fat influences bone both positively and negatively by secreting Leptin [11] and Adiponectin [12], respectively. Recently, it has been emphasized the function of brown adipocytes which also affect bone tissue byproducing factors that may be secreted to circulation or act directly in the bone marrow environment to induce osteoblast differentiation and osteocyte support for bone formation and bone turnover. Two of these factors, insulin-like growth factor binding protein 2 (IGFBP2) and wingless related MMTV integration site 10b (WNT10b), gather considerable interest because they regulate both bone remodelling and energy metabolism [13]. Moreover, beside its classical functions, bone acts in turn as endocrine organ secreting Osteocalcin, a hormone active on glucose and fat metabolism, stimulating insulin secretion and *β*-cell proliferation [14]. Of further significance, the discovering of Irisin, which is released from muscle, acts as endocrine molecule targeting adipose tissue by increasing energy expenditure [3] and bone by enhancing osteoblast differentiation. As shown in this work, Irisin is a new protagonist of the axis, which now could be considered as the muscle-fat-bone axis.

This growing body of evidences suggests a functional fat-bone axis [30], but the discovery of Irisin, together with our new findings, add a new protagonist to this axis, allowing enlarging it as the muscle-fat-bone axis (Figure 5).

Based on the scenario described, in which both muscle and BAT affect bone metabolism, it might be questioning whether the effect of physical activity on the skeleton could also be mediated by BAT, which in turn has been affected by Irisin. This would imply double, direct and indirect, Irisin action on bone *in vivo*. In our hands the Irisin-dependent action on osteoblasts is further supported by the increase of ALP and Collagen I expression, observed in osteoblasts cultured in the presence of CM from muscle cells of wheel mice. The Irisin involvement was proved by the fact that the CM-induced upregulation of ALP and Collagen I was abolished when cells were treated with CM containing anti-Irisin antibody. This suggests that Irisin does not target only adipocytes but also other body compartments, according to recent data demonstrating that Irisin could play a role in the central nervous system. In this context, recent studies revealed that cerebellar Purkinje cells of rat and mice express Irisin [31], which is also required for the proper neural differentiation of mouse embryonic stem cells [32]. Hence, given that physical activity improves neurogenesis, by reducing risk of neurodegenerative diseases such as Alzheimer and Parkinson [33, 34], Irisin could represent the molecular link between exercise and healthy brain.

## 5. Conclusions

In conclusion, we showed a novel role of Irisin, adding new evidence to the complex muscle-fat-bone axis (Figure 5). This seems remarkably promising, considering the aforementioned tight relationships between skeletal muscle and bone. Our future efforts will focus on a deepen analysis of the molecular signalling triggered by its action and, in particular, on the overall effect of Irisin in the bone context. Moreover, the characterization of its receptor will allow a more clear understanding of Irisin-induced signalling. In this respect, we would also elucidate whether this myokine affects osteoclasts, the bone resorbing cells. This might better explain the global regulation of skeletal homeostasis exerted by physical exercise or, conversely, by lack of mechanical loading.

Future studies could reveal whether expectations on the potential role of Irisin as pharmacological treatment will be confirmed. Hopefully, with regard to the skeleton, Irisin could represent a new anabolic therapy to gain bone mass in osteopenia caused by muscle-disabling diseases, such as sarcopenia, tumor-associated cachexia, neuromuscular disease, or situations with forced lack of mechanical loading, such as absence of gravity which astronauts undergo.

## References

[1] Dunstan D (2011). Diabetes: exercise and T2DM-move muscles more often!. *Nature Reviews Endocrinology*.

[2] Crepaldi G, Maggi S (2005). Sarcopenia and osteoporosis: a hazardous duet. *Journal of Endocrinological Investigation*.

[3] Boström P, Wu J, Jedrychowski MP (2012). A PGC1-*α*-dependent myokine that drives brown-fat-like development of white fat and thermogenesis. *Nature*.

[4] LeBlanc AD, Spector ER, Evans HJ, Sibonga JD (2007). Skeletal responses to space flight and the bed rest analog: a review. *Journal of Musculoskeletal Neuronal Interactions*.

[5] Karasik D, Kiel DP (2010). Evidence for pleiotropic factors in genetics of the musculoskeletal system. *Bone*.

[6] Jones HH, Priest JD, Hayes WC (1977). Humeral hypertrophy in response to exercise. *Journal of Bone and Joint Surgery A*.

[7] Rodriguez JI, Palacios J, Garcia-Alix A, Pastor I, Paniagua R (1988). Effects of immobilization on fetal bone development. A morphometric study in newborns with congenital neuromuscular diseases with intrauterine onset. *Calcified Tissue International*.

[8] Ralis ZA, Ralis HM, Randall M (1976). Changes in shape, ossification and quality of bones in children with spina bifida. *Developmental Medicine and Child Neurology*.

[9] Robling AG, Turner CH (2009). Mechanical signaling for bone modeling and remodeling. *Critical Reviews in Eukaryotic Gene Expression*.

[10] Han Y, Cowin SC, Schaffler MB, Weinbaum S (2004). Mechanotransduction and strain amplification in osteocyte cell processes. *Proceedings of the National Academy of Sciences of the United States of America*.

[11] Burguera B, Hofbauer LC, Thomas T (2001). Leptin reduces ovariectomy-induced bone loss in rats. *Endocrinology*.

[12] Kajimura D, Lee HW, Riley KJ (2013). Adiponectin regulates bone mass via opposite central and peripheral mechanisms through FoxO1. *Cell Metabolism*.

[13] Cannon B, Nedergaard J (2010). Metabolic consequences of the presence or absence of the thermogenic capacity of brown adipose tissue in mice (and probably in humans). *International Journal of Obesity*.

[14] Lee NK, Sowa H, Hinoi E (2007). Endocrine regulation of energy metabolism by the skeleton. *Cell*.

[15] Ferrer-Martínez A, Ruiz-Lozano P, Chien KR (2002). Mouse PeP: a novel peroxisomal protein linked to myoblast differentiation and development. *Developmental Dynamics*.

[16] Pedersen BK, Akerström TC, Nielsen AR, Fischer CP (2007). Role of myokines in exercise and metabolism. *Journal of Applied Physiology*.

[17] Tisdale MJ (2009). Mechanisms of cancer cachexia. *Physiological Reviews*.

[18] Filippin LI, Teixeira VN, Viacava PR, Lora PS, Xavier LL, Xavier RM (2013). Temporal development of muscle atrophy in murine model of arthritis is related to disease severity. *Journal of Cachexia, Sarcopenia and Muscle*.

[19] Timmons JA, Baar K, Davidsen PK, Atherton PJ (2012). Is irisin a human exercise gene?. *Nature*.

[20] Huh JY, Panagiotou G, Mougios V (2012). FNDC5 and irisin in humans: I. Predictors of circulating concentrations in serum and plasma and II. mRNA expression and circulating concentrations in response to weight loss and exercise. *Metabolism*.

[21] Speakman JR, Selman C (2003). Physical activity and resting metabolic rate. *Proceedings of the Nutrition Society*.

[22] Frontini A, Cinti S (2010). Distribution and development of brown adipocytes in the murine and human adipose organ. *Cell Metabolism*.

[23] Cypess AM, Lehman S, Williams G (2009). Identification and importance of brown adipose tissue in adult humans. *The New England Journal of Medicine*.

[24] Van Marken Lichtenbelt WD, Vanhommerig JW, Smulders NM (2009). Cold-activated brown adipose tissue in healthy men. *The New England Journal of Medicine*.

[25] Virtanen KA, Lidell ME, Orava J (2009). Functional brown adipose tissue in healthy adults. *The New England Journal of Medicine*.

[26] Saito M, Okamatsu-Ogura Y, Matsushita M (2009). High incidence of metabolically active brown adipose tissue in healthy adult humans: effects of cold exposure and adiposity. *Diabetes*.

[27] Bredella MA, Fazeli PK, Freedman LM (2012). Young women with cold-activated brown adipose tissue have higher bone mineral density and lower Pref-1 than women without brown adipose tissue: a study in women with anorexia nervosa, women recovered from anorexia nervosa, and normal-weight women. *Journal of Clinical Endocrinology and Metabolism*.

[28] Rahman S, Lu Y, Czernik PJ, Rosen CJ, Enerback S, Lecka-Czernik B (2013). Inducible brown adipose tissue, or beige fat, is anabolic for the skeleton. *Endocrinology*.

[29] Motyl KJ, Bishop KA, DeMambro VE (2013). Altered thermogenesis and impaired bone remodeling in Misty mice. *Journal of Bone and Mineral Research*.

[30] Kawai M, de Paula FJ, Rosen CJ (2012). New insights into osteoporosis: the bone-fat connection. *Journal of Internal Medicine*.

[31] Dun SL, Lyu RM, Chen YH, Chang JK, Luo JJ, Dun NJ (2013). Irisin-immunoreactivity in neural and non-neural cells of the rodent. *Neuroscience*.

[32] Hashemi MS, Ghaedi K, Salamian A (2013). Fndc5 knockdown significantly decreased neural differentiation rate of mouse embryonic stem cells. *Neuroscience*.

[33] Mattson MP (2012). Energy intake and exercise as determinants of brain health and vulnerability to injury and disease. *Cell Metabolism*.

[34] Erickson KI, Weinstein AM, Lopez OL (2012). Physical activity, brain plasticity, and Alzheimer’s disease. *Archives of Medical Research*.

